# Another small molecule in the oncometabolite mix: L-2-Hydroxyglutarate in kidney cancer

**DOI:** 10.18632/oncoscience.165

**Published:** 2015-05-15

**Authors:** Eun-Hee Shim, Sunil Sudarshan

**Affiliations:** ^1^ Department of Urology, University of Alabama at Birmingham, Birmingham, AL, United States

**Keywords:** renal cancer, 2-hydroxyglutarate, L2HGDH, 5-hydroxymethylcytosine, epigenetics

## Abstract

Alterations in metabolism are now considered a hallmark of cancer. One of the clearest links between metabolism and malignancy are oncometabolites. To date, several putative oncometabolites with transforming properties have been identified in the context of tumors due to both gain and loss of function mutations in genes encoding enzymes of intermediary metabolism. Through an unbiased metabolomics approach, we identified elevations of the metabolite 2-hydroxyglutarate (2-HG) in the most common histology of kidney cancer that is among the most common malignancies in both men and women. Subsequent analyses demonstrate that the predominant enantiomer of 2-HG elevated in renal cancer is the L(S) form. Notably, elevations of L-2HG are due in part to loss of expression of the L-2HG dehydrogenase (L2HGDH) which normally serves as an enzyme of “metabolite repair” to keep levels of this metabolite from accumulating. Lowering L-2HG levels in RCC through re-expression of L2HGDH mitigates tumor phenotypes and reverses epigenetic alterations known to be targeted by oncometabolites. These data add to the growing body of evidence that metabolites, similarly to oncogenes and oncoproteins, can play a role in tumor development and/or progression. As such, they represent a unique opportunity to utilize these findings in the clinic setting.

## INTRODUCTION

Cancer-associated mutations in genes encoding key metabolic enzymes have established a direct link between altered metabolism and cancer. Loss-of-function mutations in genes encoding tricarboxylic acid (TCA) cycle enzymes fumarate hydratase (*FH*) and succinate dehydrogenase (*SDH*) lead to accumulation of fumarate and succinate, respectively, whereas gain-of-function mutations in isocitrate dehydrogenase (*IDH1/2*) cause increased levels of D-2-hydroxyglutarate (D-2HG) [1-4]. Mutant IDH1/2 forms a dimer with the wild-type IDH and obtains a neomorphic activity to catalyze the reduction of α-ketoglutarate (α-KG) directly to D-2HG in the presence of NADPH. This chiral molecule is structurally similar to α-KG and is normally present at low levels in both its D-/L-enantiomers [5]. The abnormal accumulation of these oncometabolites inhibits histone demethylation and DNA hydroxylation by competitively inhibiting the catalytic activities of α-KG-dependent enzymes including the Junomji histone demethylases and ten-eleven translocation enzymes (TETs 1-3), respectively [6,7]. The TETs convert 5-methylcytosine (5-mC) to 5-hydroxymethylcytosine (5-hmC), a reaction though to promote DNA demethylation via passive and/or active means. However, recent studies in acute myelogenous leukemia suggest that 5hmC may be a stable epigenetic mark [8]. Somatic mutations in *IDH1* and *IDH2* have been demonstrated in many human cancers including low grade glioma, glioblastoma, cholangiocarcinoma, chondrosarcoma, and acute myeloid leukemia as recently summarized [5].

The L-enantiomer of 2HG (L-2HG) is generally more potent at inhibiting α-KG-dependent dioxygenases as demonstrated by cell-free and *in vitro* studies [6,7,9]. In contrast to D-2HG produced by *IDH* mutations, L-2HG is likely formed from α-KG through the “off-target” activity of malate dehydrogenase (MDH) [10]. L-2HG dehydrogenase (L2HGDH) is present to counter this off-target reaction by converting L-2HG back to α-KG. As such, L2HGDH is referred to as an enzyme of metabolite repair [11]. L-2HG has previously been linked to L-2-hydroxyglutaric aciduria (L-2HGA), a rare metabolic disorder caused by a defect in L2HGDH[12]. Intriguingly, brain tumors have been described in several L-2HGA cohorts from distinct geographical regions [13-17]. Despite these data, a clear connection between L-2HG and cancer remained to be established until our recent findings in kidney cancer [18]. We performed nontargeted metabolomics profiling in clear-cell renal cell carcinoma (ccRCC-the most common histology) and matched normal kidney. This pairwise analysis identified elevations of 2-HG in ccRCC. Subsequent enantiomeric resolution via tandem liquid chromatography-mass spectrometry (LC-MS) demonstrated that the predominant form of 2-HG elevated in ccRCC was the L(*S*) enantiomer in contrast to *IDH* mutant tumors. Interestingly, we found that elevated L-2HG tumors had reduced DNA 5-hmC levels compared with normal kidney and low L-2HG tumor consistent with the ability of this molecule to block TET activity. Consistent with prior studies, treatment of untransformed renal epithelial cells with cell-permeable L-2HG octyl ester treatment inhibited TET activity and reduced 5hmC levels. The elevations of L-2HG prompted us to examine L2HGDH expression. We analyzed L2HGDH expression in high L-2HG ccRCC tumors and RCC cell lines and confirmed that L-2HG levels are inversely correlated with L2HGDH expression. Notably, the *L2HGDH* locus is located at 14q, a region commonly lost in ccRCC and associated with worsened patient outcomes [19,20]. Knockdown or ectopic expression of L2HGDH modulated DNA hydroxymethylation and histone methylation in RCC cell lines. Furthermore, L2HGDH reconstitution in RCC cell lines suppressed *in vitro* tumor phenotypes. Together, these data suggest that L2HGDH has metabolic tumor suppressor activity and is an epigenetic regulator in kidney cancer.

Despite the relatively recent connection between *IDH* mutations and D-2HG, several clinical implications are emerging. In the context of glioma, the presence of *IDH* mutation confers a better prognosis for patients [21,22]. Moreover, recent studies indicate that D-2HG can be detected via magnetic resonance imaging (MRI)-based spectroscopy of the brain[23]. In the context of leukemia and cholangiocarcinoma, D-2HG may have the potential to be used as a biomarker given that it can be detected in the serum of patients with *IDH* mutant tumors [24,25]. Preclinical studies demonstrate the efficacy of inhibitors of mutant IDH for the treatment of leukemia. Indeed, small-molecule inhibitors against mutant forms of IDH1 and IDH2 have demonstrated reduction of D-2HG levels and led to increased differentiation and/or growth suppression of tumor cells [26,27]. This has led to the introduction of such agents into clinical trials. While *IDH* mutation is the most well characterized mechanism for D-2HG elevations in cancer, alternate mechanisms may promote D-2HG accumulation in malignancy. Phosphoglycerate dehydrogenase (PHGDH), an enzyme involved in the *de novo* synthesis of serine, has recently been shown to catalyze the NADH-dependent reduction of α-KG to D-2HG [28]. These findings are significant as amplification of PHGDH (located on chromosome 1p12) occurs in about 16% of all human cancers, including 6% of breast cancers and 40% of melanomas [29]. PHGDH overexpression in breast epithelial cells enhances the acquisition of malignant properties, whereas silencing of PHGDH inhibits growth of PHGDH-amplified cells identifying PHGDH as a potential therapeutic target in tumors. Whether the effects of PHGDH on tumorigenesis are D-2HG dependent remains to be determined. In addition, MYC overexpression in breast cancer promotes 2-HG accumulation in a glutamine dependent manner [30]. Our results demonstrate that L-2HG is elevated in kidney cancer adds another layer of complexity to 2HG in cancer. Routine LC-MS analytical methods to detect 2HG do not distinguish between D-2HG and L-2HG [31]. Therefore, it is imperative to distinguish between these forms as there are distinct biochemical pathways for each enantiomer that mediate both synthesis as well as metabolism. Thus it appears that increases in 2HG levels (either D- or L-) can occur in cancer as the result of alteration in several genes (Table 1). If in fact 2-HG is tumor promoting in these settings, then differential strategies may be employed based on the underlying mechanism of 2-HG elevation. In addition, the chiral nature of 2-HG indicates that there are likely enantiomer-specific targets, which remains largely unexplored. Further research into how prevailing the L-2HG oncometabolite is in cancer and the identification of biologically significant targets is of paramount importance. Such studies should bolster the notion of cancer metabolism as a rational target for therapeutic approaches.

**Table 1:** Oncometabolite-related enzymes and associated tumor types

Enzyme	2-HG enantiomer	Tumor	Ref.
IDH1	D-2HG	AML Glioma Secondary Glioblastomas Chondrosarcoma Cholangiocarcinoma Melanoma Prostate cancer	Mardis *et al*. 2009 Yan *et al*. 2009 Yan *et al*. 2009 Amary *et al*. 2011 Borger *et al*. 2012 Shibata *et al*. 2011 Kang *et al*. 2009
IDH2	D-2HG	AML Glioma Secondary Glioblastomas Chondrosarcoma Cholangiocarcinoma Angioimmunoblastic T-Cell Lymphomas (Aitls)	Ward *et al*. 2010 Yan *et al*. 2009 Yan *et al*. 2009 Amary *et al*. 2011 Borger *et al*. 2012 Cairns *et al*. 2012
PHGDH	D-2HG	Breast Cancer Cells	Fan *et al*. 2015
MYC	not specified	Breast Cancer	Terunuma *et al*. 2014
L2HGDH	L-2HG	Renal Cancer	Shim *et al*. 2014

## References

[1] Dang L, White DW, Gross S, Bennett BD, Bittinger MA, Driggers EM, Fantin VR, Jang HG, Jin S, Keenan MC, Marks KM, Prins RM, Ward PS, Yen KE, Liau LM, Rabinowitz JD (2009). Cancer-associated IDH1 mutations produce 2-hydroxyglutarate. Nature.

[2] Gross S, Cairns RA, Minden MD, Driggers EM, Bittinger MA, Jang HG, Sasaki M, Jin S, Schenkein DP, Su SM, Dang L, Fantin VR, Mak TW (2010). Cancer-associated metabolite 2-hydroxyglutarate accumulates in acute myelogenous leukemia with isocitrate dehydrogenase 1 and 2 mutations. J Exp Med.

[3] Pollard PJ, Briere JJ, Alam NA, Barwell J, Barclay E, Wortham NC, Hunt T, Mitchell M, Olpin S, Moat SJ, Hargreaves IP, Heales SJ, Chung YL, Griffiths JR, Dalgleish A, McGrath JA (2005). Accumulation of Krebs cycle intermediates and over-expression of HIF1alpha in tumours which result from germline FH and SDH mutations. Human molecular genetics.

[4] Ward PS, Patel J, Wise DR, Abdel-Wahab O, Bennett BD, Coller HA, Cross JR, Fantin VR, Hedvat CV, Perl AE, Rabinowitz JD, Carroll M, Su SM, Sharp KA, Levine RL, Thompson CB (2010). The common feature of leukemia-associated IDH1 and IDH2 mutations is a neomorphic enzyme activity converting alpha-ketoglutarate to 2-hydroxyglutarate. Cancer Cell.

[5] Cairns RA, Mak TW (2013). Oncogenic isocitrate dehydrogenase mutations: mechanisms, models, and clinical opportunities. Cancer Discov.

[6] Chowdhury R, Yeoh KK, Tian YM, Hillringhaus L, Bagg EA, Rose NR, Leung IK, Li XS, Woon EC, Yang M, McDonough MA, King ON, Clifton IJ, Klose RJ, Claridge TD, Ratcliffe PJ (2011). The oncometabolite 2-hydroxyglutarate inhibits histone lysine demethylases. EMBO Rep.

[7] Xu W, Yang H, Liu Y, Yang Y, Wang P, Kim SH, Ito S, Yang C, Xiao MT, Liu LX, Jiang WQ, Liu J, Zhang JY, Wang B, Frye S, Zhang Y (2011). Oncometabolite 2-hydroxyglutarate is a competitive inhibitor of alpha-ketoglutarate-dependent dioxygenases. Cancer Cell.

[8] Rampal R, Alkalin A, Madzo J, Vasanthakumar A, Pronier E, Patel J, Li Y, Ahn J, Abdel-Wahab O, Shih A, Lu C, Ward PS, Tsai JJ, Hricik T, Tosello V, Tallman JE (2014). DNA hydroxymethylation profiling reveals that WT1 mutations result in loss of TET2 function in acute myeloid leukemia. Cell reports.

[9] Koivunen P, Lee S, Duncan CG, Lopez G, Lu G, Ramkissoon S, Losman JA, Joensuu P, Bergmann U, Gross S, Travins J, Weiss S, Looper R, Ligon KL, Verhaak RG, Yan H (2012). Transformation by the (R)-enantiomer of 2-hydroxyglutarate linked to EGLN activation. Nature.

[10] Rzem R, Vincent MF, Van Schaftingen E, Veiga-da-Cunha M (2007). L-2-hydroxyglutaric aciduria, a defect of metabolite repair. J Inherit Metab Dis.

[11] Van Schaftingen E, Rzem R, Marbaix A, Collard F, Veiga-da-Cunha M, Linster CL (2013). Metabolite proofreading, a neglected aspect of intermediary metabolism. J Inherit Metab Dis.

[12] Rzem R, Veiga-da-Cunha M, Noel G, Goffette S, Nassogne MC, Tabarki B, Scholler C, Marquardt T, Vikkula M, Van Schaftingen E (2004). A gene encoding a putative FAD-dependent L-2-hydroxyglutarate dehydrogenase is mutated in L-2-hydroxyglutaric aciduria. Proceedings of the National Academy of Sciences of the United States of America.

[13] Aghili M, Zahedi F, Rafiee E (2009). Hydroxyglutaric aciduria and malignant brain tumor: a case report and literature review. J Neurooncol.

[14] Barbot C, Fineza I, Diogo L, Maia M, Melo J, Guimaraes A, Pires MM, Cardoso ML, Vilarinho L (1997). L-2-Hydroxyglutaric aciduria: clinical, biochemical and magnetic resonance imaging in six Portuguese pediatric patients. Brain Dev.

[15] Haliloglu G, Jobard F, Oguz KK, Anlar B, Akalan N, Coskun T, Sass JO, Fischer J, Topcu M (2008). L-2-hydroxyglutaric aciduria and brain tumors in children with mutations in the L2HGDH gene: neuroimaging findings. Neuropediatrics.

[16] Moroni I, Bugiani M, D'Incerti L, Maccagnano C, Rimoldi M, Bissola L, Pollo B, Finocchiaro G, Uziel G (2004). L-2-hydroxyglutaric aciduria and brain malignant tumors: a predisposing condition?. Neurology.

[17] Ozisik PA, Akalan N, Palaoglu S, Topcu M (2002). Medulloblastoma in a child with the metabolic disease L-2-hydroxyglutaric aciduria. Pediatr Neurosurg.

[18] Shim EH, Livi CB, Rakheja D, Tan J, Benson D, Parekh V, Kho EY, Ghosh AP, Kirkman R, Velu S, Dutta S, Chenna B, Rea SL, Mishur RJ, Li Q, Johnson-Pais TL (2014). L-2-Hydroxyglutarate: an epigenetic modifier and putative oncometabolite in renal cancer. Cancer Discov.

[19] Kroeger N, Klatte T, Chamie K, Rao PN, Birkhauser FD, Sonn GA, Riss J, Kabbinavar FF, Belldegrun AS, Pantuck AJ (2013). Deletions of chromosomes 3p and 14q molecularly subclassify clear cell renal cell carcinoma. Cancer.

[20] Monzon FA, Alvarez K, Peterson L, Truong L, Amato RJ, Hernandez-McClain J, Tannir N, Parwani AV, Jonasch E (2011). Chromosome 14q loss defines a molecular subtype of clear-cell renal cell carcinoma associated with poor prognosis. Modern pathology: an official journal of the United States and Canadian Academy of Pathology, Inc.

[21] Parsons DW, Jones S, Zhang X, Lin JC, Leary RJ, Angenendt P, Mankoo P, Carter H, Siu IM, Gallia GL, Olivi A, McLendon R, Rasheed BA, Keir S, Nikolskaya T, Nikolsky Y (2008). An integrated genomic analysis of human glioblastoma multiforme. Science.

[22] Yan H, Parsons DW, Jin G, McLendon R, Rasheed BA, Yuan W, Kos I, Batinic-Haberle I, Jones S, Riggins GJ, Friedman H, Friedman A, Reardon D, Herndon J, Kinzler KW, Velculescu VE (2009). IDH1 and IDH2 mutations in gliomas. N Engl J Med.

[23] Choi C, Ganji SK, DeBerardinis RJ, Hatanpaa KJ, Rakheja D, Kovacs Z, Yang XL, Mashimo T, Raisanen JM, Marin-Valencia I, Pascual JM, Madden CJ, Mickey BE, Malloy CR, Bachoo RM, Maher EA (2012). 2-hydroxyglutarate detection by magnetic resonance spectroscopy in IDH-mutated patients with gliomas. Nature medicine.

[24] Borger DR, Goyal L, Yau T, Poon RT, Ancukiewicz M, Deshpande V, Christiani DC, Liebman HM, Yang H, Kim H, Yen K, Faris JE, Iafrate AJ, Kwak EL, Clark JW, Allen JN (2014). Circulating oncometabolite 2-hydroxyglutarate is a potential surrogate biomarker in patients with isocitrate dehydrogenase-mutant intrahepatic cholangiocarcinoma. Clin Cancer Res.

[25] Fathi AT, Sadrzadeh H, Borger DR, Ballen KK, Amrein PC, Attar EC, Foster J, Burke M, Lopez HU, Matulis CR, Edmonds KM, Iafrate AJ, Straley KS, Yen KE, Agresta S, Schenkein DP (2012). Prospective serial evaluation of 2-hydroxyglutarate, during treatment of newly diagnosed acute myeloid leukemia, to assess disease activity and therapeutic response. Blood.

[26] Wang F, Travins J, DeLaBarre B, Penard-Lacronique V, Schalm S, Hansen E, Straley K, Kernytsky A, Liu W, Gliser C, Yang H, Gross S, Artin E, Saada V, Mylonas E, Quivoron C (2013). Targeted inhibition of mutant IDH2 in leukemia cells induces cellular differentiation. Science.

[27] Rohle D, Popovici-Muller J, Palaskas N, Turcan S, Grommes C, Campos C, Tsoi J, Clark O, Oldrini B, Komisopoulou E, Kunii K, Pedraza A, Schalm S, Silverman L, Miller A, Wang F (2013). An inhibitor of mutant IDH1 delays growth and promotes differentiation of glioma cells. Science.

[28] Fan J, Teng X, Liu L, Mattaini KR, Looper RE, Vander Heiden MG, Rabinowitz JD (2015). Human Phosphoglycerate Dehydrogenase Produces the Oncometabolite d-2-Hydroxyglutarate. ACS chemical biology.

[29] Locasale JW, Grassian AR, Melman T, Lyssiotis CA, Mattaini KR, Bass AJ, Heffron G, Metallo CM, Muranen T, Sharfi H, Sasaki AT, Anastasiou D, Mullarky E, Vokes NI, Sasaki M, Beroukhim R (2011). Phosphoglycerate dehydrogenase diverts glycolytic flux and contributes to oncogenesis. Nature genetics.

[30] Terunuma A, Putluri N, Mishra P, Mathe EA, Dorsey TH, Yi M, Wallace TA, Issaq HJ, Zhou M, Killian JK, Stevenson HS, Karoly ED, Chan K, Samanta S, Prieto D, Hsu TY (2014). MYC-driven accumulation of 2-hydroxyglutarate is associated with breast cancer prognosis. J Clin Invest.

[31] Struys EA (2013). 2-Hydroxyglutarate is not a metabolite; D-2-hydroxyglutarate and L-2-hydroxyglutarate are!. Proceedings of the National Academy of Sciences of the United States of America.

